# Breast Cancer Screening in Transgender Population: Review of literature

**DOI:** 10.12669/pjms.40.8.9132

**Published:** 2024-09

**Authors:** Saffa Tareen, Mehwish Mooghal, Kulsoom Shaikh, Sana Zeeshan

**Affiliations:** 1 Saffa Tareen, MBBS Resident Surgery, Department of Surgery, The Aga Khan University, Karachi, Pakistan; 2 Mehwish Mooghal, FCPS Fellow Breast Surgery, Department of Surgery, The Aga Khan University, Karachi, Pakistan; 3 Kulsoom Shaikh, FCPS Fellow Breast Surgery, Department of Surgery, The Aga Khan University, Karachi, Pakistan; 4 Sana Zeeshan, FCPS, FACS Assistant Professor Breast Surgery, Department of Surgery, The Aga Khan University, Karachi, Pakistan

**Keywords:** Transgenders, Mass Screening, Healthcare Disparities, Cultural Competency, Breast Cancer

## Abstract

This literature review explores breast cancer screening practices among transgender individuals globally, emphasizing the overlooked population in Pakistan. With an overview of intersex and transgender terminology, the study delves into screening guidelines for transfeminine and transmasculine patients, considering hormone therapy and surgery. Worldwide statistics on transgender and intersex populations are provided, highlighting the unique challenges they face, particularly in Pakistan, where societal discrimination and healthcare barriers persist.

Databases searched included PubMed, Scopus, and Google Scholar from the Year 2000 till todate.The review synthesizes breast cancer screening recommendations in transgender population from ACR, WPATH, UCSF, and the Canadian Cancer Society, revealing variations in guidelines. It concludes with a call for tailored screening protocols for Pakistan’s transgender community and recommends a comprehensive study due to the absence of data in Southeast Asia. The unstructured abstract underscores the need for nuanced, personalized screening strategies and emphasizes the critical gap in knowledge specific to breast cancer in this marginalized population.

## INTRODUCTION

Intersex is an emerging umbrella term that encompasses medically recognized conditions that result from natural variation of chromosome combination, hormone balance or internal and external body parts in a person formerly referred to as Disorders of Sexual Development (DSD).^1^ Transgender is a generalized term for any individual whose gender identity, or internal sense of self-related to gender, differs from the sex assigned at birth.^1^ A nonbinary individual may have been assigned as female or male at birth but does not strictly identify with either sex.^1^

A transfeminine person identifies with the female side of the gender spectrum but was assigned male at birth. These individuals may include transgender women, historically referred to as male-to-female transsexuals.^2^ A transmasculine person identifies with the male side of the gender spectrum but was assigned female at birth. These individuals may include transgender men, historically referred to as female-to-male transsexuals.^2^ Worldwide, 1% of the population is transgender and it is estimated that up to 1.7% of the population has an intersex trait and that approximately 0.5% of people have clinically identifiable sexual or reproductive variations.^3^ According to a report published in 2022, almost 1.64 million people over the age of 13 years in the United States (U.S.) are identified as transgender having a different gender identity than the one assigned at birth.^4^

In Pakistan, the National Database officially recorded a total of > 10,000 transgender individuals. These numbers were distributed across various regions, with Punjab being the most populous, constituting 64.39% of the total, which corresponds to 6,709 transgender individuals.^5^,^6^

Around 55,900 new breast cancer cases are reported in the UK every year, which equates to more than 150 cases every day and about 55,000 new cases in females and 370 new cases in males every year.^7^ The Global Cancer Observatory (GCO) database estimates 2,261,419 new breast cancer cases and 684,996 related deaths in 2020.^8^ The overall incidence of breast cancer for transwomen and transmen combined was 43/100,000 person years due to use of Cross-sex hormones (CSHs) for psychological and physiological effect.^9^ The overall incidence was 20/100,000 patient years regardless of hormone exposure, which was not higher than the expected rate.^10^ For transgender and gender-nonconforming individuals, breast cancer screening recommendations are based on the sex assigned at birth, risk factors, and use of exogenous hormones.^1^

Understanding the unique experiences and needs of the transgender population is crucial for delivering equitable and patient-centered healthcare. This applies to screening for breast cancer also as this specific population may be at increased risk due to prolonged use of exogenous hormones. This review aimed to consolidate the current knowledge on breast cancer screening practices among transgender individuals around the world. By critically assessing the available literature, this review will explore various aspects, including screening guidelines, screening modalities, timing, and adherence rates among transgender individuals.

Additionally, the review will investigate the impact of gender-affirming interventions, such as hormone therapy and chest surgery, on breast cancer risk and the implications for screening. By evaluating the existing evidence, this review will contribute to filling the gaps in knowledge surrounding breast cancer screening in transgender individuals. The findings will shed light on the effectiveness, feasibility, and appropriateness of current screening practices and provide insights for future research and clinical guidelines specifically for the transgender population of Pakistan which is currently the most overlooked set of individuals when it comes to delivery of health care. In conclusion, it aims to inform healthcare providers, policymakers, and researchers about the current state of knowledge in this area and provide a foundation for the development of tailored screening guidelines and interventions.

## METHODS

This literature review employed a systematic approach to gather and analyze information related to breast cancer screening practices among transgender individuals globally, with a specific focus on the situation in Pakistan. A comprehensive search of electronic databases, including PubMed, Scopus, and Google Scholar, was conducted to identify relevant articles published from the year 2000 up to the present date. The search strategy incorporated keywords such as “transgender,” “breast cancer,” “screening guidelines,” and “Pakistan.” The inclusion criteria comprised peer-reviewed articles, reviews, and guidelines addressing breast cancer screening practices in transgender populations. Additionally, grey literature and reports from reputable health organizations were considered to capture a broad spectrum of information.

The identified literature was then meticulously reviewed, and relevant data was extracted. The data extraction process focused on key aspects, including screening guidelines, modalities, timing, adherence rates, and the impact of gender-affirming interventions on breast cancer risk. The selected studies were critically appraised for methodological quality and relevance to the research objectives. To address the specific context of Pakistan, additional searches were conducted for literature pertaining to the transgender population in the region, including population census, cultural aspects, healthcare access, and societal challenges. Information on the prevalence of breast cancer, screening practices, and healthcare barriers within the transgender community in Pakistan was gathered from available sources, including census reports, surveys, and academic studies.

The synthesis of findings involved a thematic analysis to categorize information according to screening guidelines for transfeminine and transmasculine individuals. Comparative analyses were performed to understand variations in recommendations from different international guidelines, such as those from American College of Radiology (ACR)^11^, World Professional Association for Transgender Health (WPATH)^12^, University of California, San Francisco (UCSF)^13^, and Canadian Cancer Society.^14^

The limitations of this review include the potential bias in the available literature and the absence of specific data on breast cancer screening for transgender individuals in Pakistan. Despite these challenges, the review aimed to provide a comprehensive overview of the existing knowledge and highlight the critical gaps in understanding breast cancer screening practices in this marginalized population. The findings from this review are expected to contribute valuable insights for future research, clinical guidelines, and healthcare interventions tailored to the transgender population in Pakistan.

### Screening Guidelines for Transgenders from across the world

There are differences in cancer-specific outcomes among transgender populations because of several factors, including increased cancer specific risk, late-stage presentation, restricted access to healthcare, stigmatisation, marginalization, and inadequate patient-provider communication, which frequently results in lower-quality care. There is a persistent knowledge gap in how to address these issues because there is a lack of information on the causes of these factors at both the individual and population levels.^15^ A 2019 nationwide cohort study conducted in the Netherlands concluded that breast cancer screening guidelines for transgender and gender-nonconforming individuals are influenced by various factors, including the assigned sex at birth, hormone treatment history (≥ 5 years), and individual risk profiles.^9^

In general, the transfeminine patients (a transgender person whose gender identity is girl/woman/female but whose sex assigned at birth was male) are commonly administered antiandrogens and estrogens, while transmasculine patients (a transgender person whose gender identity is boy/man/male but whose sex assigned at birth was female) typically receive testosterone as part of their hormone therapy.^16^ The gender (male or female) given to a newborn is typically determined by the newborn’s physical anatomy/genitalia and other genetic characteristics.^17^

Similarly, gender-affirming breast surgeries are performed for transgender individuals, with different procedures tailored to each gender identity. Transgender men may undergo reduction mammoplasty or mastectomy, commonly referred to as “top surgery,” whereas transgender women may opt for breast augmentation using implants or autologous fat grafting. The type of surgery opted by an individual also modify the screening guidelines for these patients.^15^

Regarding breast cancer screening, the literature provides specific recommendations for different patient groups. Broadly, two categories are relevant to address: Transfeminine (male to female) and Transmasculine (female to male).

### Transfeminine

Several studies have reported higher incidence of breast cancer among transfeminine cohort of patients as compared to their male counterparts who have used exogenous feminizing hormones (estrogens and antiandrogens) for more than a five years period.^10^ Another population-based study from Dutch cohort reported forty-six-fold higher incidence of breast cancer among transfeminine group when compared to their cis males.^9^ Transfeminine patients with no hormone use or less than five years of hormone use, regardless of age, are generally not considered for imaging-based breast cancer screening, as their risk of developing breast cancer is equivalent to normal male cohort.^11^,^12^

For average-risk transfeminine patients aged 40 years or older with a history of hormone use for five years or more, annual digital breast tomosynthesis (DBT) or mammography may be appropriate as per ACR guidelines.^11^ WPATH guidelines recommend their screening as per cisgender women of same age group considering the risk factors and time lapse of hormone therapy, dosing, and age.^12^ However, UCSF centre for excellence for transgender health endorses biennial screening mammography starting at the age of 50 years.^13^ Canadian Cancer society endorses two yearly screening mammograms between age 50-69 years with at least five years of hormone therapy.^14^

For higher-than-average-risk transfeminine patients (risk >20% as per risk assessment models), as characterized by family history of breast or ovarian cancer, genetic predisposition to breast cancer, untested patient with first-degree relative with genetic predisposition to breast cancer, personal history of breast cancer or chest irradiation at 10 to 30 years of age, along with hormone use for five years or more, are also recommended by ACR to undergo DBT or mammography starting by the age 25-30 years.^11^ WPATH recommends shared decision making among the high-risk cohort whereas UCSF and Canadian Cancer Society have not addressed this cohort of transfeminine population.^12^-^14^ For transfeminine patients with no hormone use or hormone use less than five years at any age, breast cancer screening is usually not recommended as the risk is equivalent to a normal male cohort.^11^,^12^ The recommendations for breast cancer screening in transfeminine individuals are summarized in Table-I.

**Table-I:** Breast Cancer Screening Recommendations for Transfeminine Patients.

Patient Group as per risk profile	Screening Guidelines	Age of initiation of screening	Duration of Hormone Use	Screening Recommendations
Transfeminine Average-risk	ACR^11^	≥ 40 years	≥ 5 years	Annual DBT or Mammography
	WPATH^12^	≥ 40 years	≥ 5 years	Annual screening as per cisgender women of the same age group
	UCSF^13^	≥ 50 years	≥ 5 years	Biennial screening mammography
	Canadian Cancer Society^14^	50-69 years	≥ 5 years	Biennial screening mammography
Transfeminine Higher-than-average-risk (Lifetime risk >20%)	ACR^13^	≥ 25 years	≥ 5 years	Annual DBT or mammography
	WPATH^12^	≥ 25 years	≥ 5 years	Shared decision-making is recommended.
	UCSF^13^	Not addressed	Not addressed	Not addressed for this specific cohort
	Canadian Cancer Society)^14^	Not addressed	Not addressed	Not addressed for this specific cohort
Transfeminine (no hormone use or < 5 years of use)^13^	ACR^11^, WPATH^12^	Not applicable	< 5 years	Generally, not considered for imaging-based breast cancer screening

ACR: American College of Radiology, WPATH: World Professional Association for Transgender Health, UCSF: University of California, San Francisco, DBT: Digital Breast Tomosynthesis.

### Transmasculine

In contrast to transfeminine individuals, transmasculine patients who have undergone bilateral mastectomies (top surgery”) are usually not recommended for breast cancer screening, as the risk of breast cancer in such cases is significantly reduced.^11^-^13^ However, for average-risk transmasculine patients aged 40 years or older with reduction mammoplasty or no chest surgery and a lifetime risk of breast cancer <15%, annual DBT or mammography starting at the age of 40 years may be considered as per guideline recommendations for cisgender females.^11^-^13^ The Canadian Cancer Society however endorses mammography every two years between age 50 and 69 years among these patients for screening.^14^

For higher-than-average-risk transmasculine patients with reduction mammoplasty or no chest surgery, annual screening mammography or DBT starting at 30 years for intermediate risk (lifetime risk of 15 to 20%) and 25–30 years for high-risk individuals (lifetime risk >20%) is recommended. In such cases, magnetic resonance imaging (MRI) of breasts with and without intravenous contrast is recommended as an adjunct to DBT or mammography for screening purposes.^11^ Intermediate risk transmasculine individuals include patients with personal history of breast cancer, lobular neoplasia and atypical ductal hyperplasia.^11^

High-risk transmasculine individuals include patient with genetic predisposition to breast cancer or untested patient with a first-degree relative with genetic predisposition to breast cancer, history of chest irradiation between 10 to 30 years of age, or with 20% or greater lifetime risk of breast cancer as per risk assessment tools.^11^ Similarly, WPATH recommends the same screening criteria for this group of population as per criteria set for higher-than-average-risk female cohort^12^, while, UCSF and Canadian Cancer Society have not commented on this group of individuals.^13^,^14^ The recommendations for breast cancer screening in transmasculine individuals are summarized in Table-II.

**Table-II:** Breast Cancer Screening Recommendations for Transmasculine Patients.

Patient Group as per risk profile	Screening Guidelines	Age of initiation of screening	Chest Surgery Status	Screening Recommendations
Transmasculine Average-risk (Lifetime risk <15%)	ACR^11^	≥ 40 years	Reduction mammoplasty or no chest surgery	Annual DBT or Mammography
	WPATH^12^	≥ 40 years	Reduction mammoplasty or no chest surgery	Annual DBT or Mammography
	UCSF^13^	≥ 40 years	Reduction mammoplasty or no chest surgery	Annual DBT or Mammography
	Canadian Cancer Society)^14^	50-69 years	Reduction mammoplasty or no chest surgery	Biennial screening mammography
Transmasculine Higher-than-average-risk (Lifetime risk >15%)	ACR^11^	≥ 30 years for intermediate risk 25–30 years for high-risk	Reduction mammoplasty or no chest surgery	Annual DBT or Mammography, consider MRI with and without intravenous contrast as an adjunct to DBT or mammography
	WPATH^12^	Not specified	Reduction mammoplasty or no chest surgery	Follow the same screening criteria as the higher-than-average-risk female cohort
	UCSF^13^	Not specified	Not specified	Not addressed for this specific cohort
	Canadian Cancer Society)^14^	Not specified	Not specified	Not addressed for this specific cohort
Transmasculine with top surgery (Significantly reduced risk)	ACR^11^ WPATH^12^UCSF^13^ Canadian Cancer Society^14^	Not applicable	Bilateral mastectomy	Not recommended for breast cancer screening

ACR: American College of Radiology, WPATH: World Professional Association for Transgender Health, UCSF: University of California, San Francisco, DBT: Digital Breast Tomosynthesis, MRI: Magnetic Resonance Imaging.

### Situation in Pakistan

In Pakistani culture, the transgender community comes from their cultural heritage of “Khawaja Sara” community who were the guardians of the ladies of Harem during Mughal times and their origin traces back to 9^th^ century BC and even earlier.^18^ Transgenders were not recognized as a separate gender in Pakistan before 2009. In 2009, transgenders were recognized as a third gender category. Their right to employment and inheritance after death of parents were recognized by the court in 2012 and Lahore High court for the first time issued the order to include transgender community in the population census.^18^

According to 2017 census, 10,418 transgenders were registered with National Database & Registration Authority (NADRA), with Punjab having 64.39% (6709), Sindh 24.25% (2527), KPK 8.73% (913), FATA 0.25% (27), Baluchistan 1.04% (109) and Islamabad 1.27% (133) of the specified population.^5^,^6^ Few unofficial surveys quote the transgender population in Pakistan to be ranging from 0.4 to 1.5 million.^19^,^20^ The transgenders are compelled to categorize themselves into gender binaries, either male or female, and failing to do so results in them being ending up with the “Gurus” who brought them up separate from their own families.^18^ They are usually not made a part of the society and are bound to live in their own colonies outside the common societies.^20^ The main customary source of income for transgenders in Pakistan include dancing, singing, prostitution and begging.^20^

Despite being given equal rights to transgenders by chief justice of Pakistan in 2009 with free education, health care and equal job opportunities, these rights seem to be only in record papers with access to the rights being alarmingly scarce as compared to their cisgender counter parts.^20^ Transgender people in Pakistan, like in many other countries, often face numerous challenges. In Pakistani culture, transgenders are recognized as symbol of shame and ignominy.^18^,^19^ The challenges commonly faced by transgender include social discrimination, violence, limited access to healthcare, education, and employment opportunities.^19^ A survey conducted on transgenders, 81% of individuals expected harassment from a doctor which prevented them from getting medical attention.^21^ The transgender community is at severe health risk due to marginalization by the families and the communities.^22^ They are at high risk of sexually transmitted infections, mental health disparities and drug abuse.^18^

A study constituting of 409 participants showed that 84% had sold sex, 94% could identify a condom, but 42% reported never needing one, 58% had sexually transmitted infections (STIs) and 38% had multiple infections.^18^ The incidence of HIV in transgender community is eight times higher as compared to the cisgender community.^23^ Another study revealed that approximately 17.5% of entire HIV population in Pakistan comprise of transgender community.^23^ A study performed in Lahore, Pakistan on 214 participants revealed only 21.5% of the transgender participants in the study were employed with majority of the study participants (57%) earning less than thirty thousand PKR per month.^24^

A significant number of study population was also seen suffering from mental health disorders with 56% of the participants suffering from depression and 59% having anxiety.^24^ Majority of the participants of the study had experienced social discrimination (89%) with 76% of the study participants being a victim of physical abuse. This study also revealed approximately 82% of study population facing neglected behaviour from health care providers with 79% facing discriminative behaviour from health care providers.^24^ Since breast cancer has a considerable prevalence among transgender population with the incidence reported at 43/100,000 person with usage of Cross-sex hormones (CSHs) and an overall incidence of 20/100,000 patient years regardless of hormone exposure,^9^,^10^ adequate guidelines and regimes are needed for breast cancer screening in transgender population in our region as well.

Unfortunately, no such data or guidelines are available for Pakistan or even for Southeast Asian transgender population related to breast cancer screening in the available published literature. The grim facts related to the living standards of transgender population of Pakistan and the barriers to healthcare as stated above limit this special population from seeking opinion for their breast health and screening. A well-planned study is required to screen the eligible transgender population of Pakistan to screen for breast cancer and if a high incidence is found, breast cancer screening guidelines for this population of Pakistan need to be developed.

### Conclusion

In conclusion, breast cancer screening recommendations for transgender and gender-nonconforming individuals involve a nuanced approach based on individual risk factors, history of hormone therapy, and surgical interventions. Clinicians must be aware of these variations to provide appropriate and personalized breast cancer screening strategies for this diverse patient population.

### Recommendations

There are no available studies or data set on breast cancer screening in this population or its prevalence or its treatment published in Pakistan or Southeast Asia so far. There is a huge gap on this topic in our part of the world and a study on this topic would make a considerable impact.

### Authors Contribution:

**ST, MM, KS:** Substantial contributions to design, drafting the article and revising it critically for important intellectual content; and final approval of the version to be published. Agreement to be accountable for all aspects of the work in ensuring that questions related to the accuracy or integrity of any part of the work are appropriately investigated and resolved.

**SZ:** Substantial contributions to conception and design, revising the article critically for important intellectual content; and final approval of the version to be published. Agreement to be accountable for all aspects of the work in ensuring that questions related to the accuracy or integrity of any part of the work are appropriately investigated and resolved.
